# Neuropsychological Decline Stratifies Dementia Risk in Cognitively Unimpaired and Impaired Older Adults

**DOI:** 10.3389/fnagi.2022.838459

**Published:** 2022-07-18

**Authors:** Jean K. Ho, Daniel A. Nation

**Affiliations:** ^1^Institute for Memory Disorders and Neurological Impairments, University of California, Irvine, Irvine, CA, United States; ^2^Department of Psychological Science, University of California, Irvine, Irvine, CA, United States

**Keywords:** mild cognitive impairment, subtle cognitive decline, dementia, Alzheimer’s disease, aging, assessment

## Abstract

**Objective:**

Validation and widespread use of markers indicating decline in serial neuropsychological exams has remained elusive despite potential value in prognostic and treatment decision-making. This study aimed to operationalize neuropsychological decline, termed “neuropsychological (NP) decline,” in older adults followed over 12 months in order to aid in the stratification of dementia risk along the cognitively unimpaired-to-mild cognitive impairment (MCI) spectrum.

**Methods:**

A prospective cohort study utilized 6,794 older adults from the National Alzheimer’s Coordinating Center (NACC) database with a baseline diagnosis of normal cognition, impaired without MCI or with MCI. Operationalization of NP decline over 12-month follow-up used regression-based norms developed in a robustly normal reference sample. The extent to which each participant’s 12-month follow-up score deviated from norm-referenced expectations was quantified and standardized to an NP decline z-score. Cox regression evaluated whether the NP decline metric predicted future dementia.

**Results:**

Participant’s NP decline scores predicted future all-cause dementia in the total sample, χ^2^ = 110.71, hazard ratio (HR) = 1.989, *p* < 0.001, and in the subset diagnosed with normal cognition, χ^2^ = 40.84, HR = 2.006, *p* < 0.001, impaired without MCI diagnosis, χ^2^ = 14.89, HR = 2.465, *p* < 0.001, and impaired with MCI diagnosis, χ^2^ = 55.78, HR = 1.916, *p* < 0.001.

**Conclusion:**

Operationalizing NP decline over 12 months with a regression-based norming method allows for further stratification of dementia risk along the cognitively unimpaired-to-MCI spectrum. The use of NP decline as an adjunctive marker of risk beyond standard cognitive diagnostic practices may aid in prognosis and clinical decision-making.

## Introduction

Early identification of older adults at risk for dementia remains an important research goal, as preventative efforts will likely require early intervention (Crous-Bou et al., 2017). Although mild cognitive impairment (MCI) is an important and useful diagnostic construct that represents an intermediate level of cognitive impairment between normal cognition and dementia (Petersen, 2011), recent research has increasingly focused on earlier stratification of dementia risk in cognitively unimpaired older adults (Amieva et al., 2005; Machulda et al., 2013; Hassenstab et al., 2015; Han et al., 2017). Efforts aimed at identifying cognitively unimpaired older adults at risk for dementia have predominantly emphasized the role of biological markers in index underlying neuropathology (Jack et al., 2018). However, numerous studies also indicate that subtle cognitive changes are detectable on a neuropsychological exam in cognitively unimpaired older adults at risk for dementia (Edmonds et al., 2015b; Han et al., 2017; Ho and Nation, 2018; Thomas et al., 2020).

There are inherent limits in the ability to establish cutoff values and diagnostic criteria for the diagnosis of subtle or mild impairments based on a single exam. Thus, longitudinal assessment of cognitive change within an individual may aid in the detection of early decline within normal range performance (Koscik et al., 2019; Nation et al., 2019). However, serial cognitive exams introduce practice effects and regression to the mean, complicating the interpretation of decline (Crawford and Howell, 1998; Slick, 2006). Nevertheless, recent studies suggest that serial cognitive performance may still be of value. For example, the lack of a practice effect may actually be indicative of a subtle cognitive decline in older adults at risk for dementia (Machulda et al., 2013; Hassenstab et al., 2015; Duff et al., 2017; Papp et al., 2020). These findings suggest the potential value of obtaining normative data on serial cognitive exam performance in older adults to supplement single exam data.

Obtaining information regarding the trajectory of cognitive change may aid efforts to refine MCI diagnostic accuracy and predictive value (Nation et al., 2019). Fluctuation in cognitive performance and reversion from MCI to normal performance across exams is common, even among individuals with underlying neuropathology (Thomas et al., 2019). If the trajectory of cognitive change was available in patients with MCI through normative comparisons of cognitive change, further characterization of MCI-associated risk could be possible.

To evaluate the predictive value of serial neuropsychological exam analysis, we previously operationalized neuropsychological decline, termed “NP decline,” over 1 year using the Alzheimer’s Disease Neuroimaging Initiative (ADNI) study (Nation et al., 2019). In this study, NP decline in cognitively unimpaired older adults, and those diagnosed with MCI, was associated with an increased risk for future clinical diagnosis of Alzheimer’s dementia. This study sought to further validate this previously developed NP decline metric and determine its predictive value for all-cause dementia. We hypothesized that, consistent with our previous results, NP decline would be predictive of future Alzheimer’s disease, even in a larger and more heterogeneous sample of 6,794 older adults from the National Alzheimer’s Coordinating Center (NACC) database.

## Methods

### National Alzheimer’s Coordinating Center Study Data and Participants

This prospective cohort study utilized longitudinal participant data obtained from the NACC database, a repository of data on aging and dementia gathered from Alzheimer’s Disease Centers (ADCs) across the country using a Uniform Data Set (UDS). The UDS includes harmonized protocols for data collection and entry regarding information from in-person visits for health and neurological examination, neuropsychological testing, and psychosocial and biological measures. In this study, NACC UDS data from the cognitive diagnostic exam and neuropsychological exam were analyzed, and all available follow-up data through December 2018 were included. The duration of available participant follow-up data varied from 18 to 156 months after baseline. Given the switch in verbal memory measures between UDS 2.0 and 3.0, we included data from Logical Memory only and did not include Craft Story data.

We limited our analysis to the 6,794 participants who were aged 60 years and older, had been diagnosed “cognitively normal,” “impaired without MCI” or “MCI,” according to the NACC UDS protocol criteria, and had been followed for at least two additional follow-up study visits extending more than 12 months from baseline. All participants needed 12-month follow-up data in order to calculate NP decline scores and needed to remain non-demented at a 12-month follow-up in order to be included in the analysis of 12-month NP decline as a predictor of future dementia. Similarly, all participants required the third evaluation after their 12-month follow-up exam in order to be evaluated in terms of the predictive value of a 12-month NP decline for the risk for future dementia. Thus, participants who progressed to dementia within 12 months of baseline, had fewer than 3 exams, or had less than ≤12 months of total follow-up were excluded.

Participants from NACC are assigned a diagnosis following adjudication by an experienced clinician or an interdisciplinary team (Morris et al., 2006). Psychosocial functioning, history, as well as test performance in various cognitive domains (recall, attention, executive function, language, and visuospatial functioning) are under consideration during these adjudications. Diagnoses in NACC are informed by neuropsychological testing, but are made clinically and are not based on strict cutoff values on these measures. Participants receive a diagnosis of (a) “cognitively normal” if they lack significant functional or cognitive impairment, (b) “MCI” if they have subjective or objective evidence of cognitive impairment without significant functional impairment, and (c) “demented” if they have both significant functional and cognitive impairment.

All contributing ADCs obtained informed consent from their participants and maintained separate IRB review and approval from their institutions prior to submitting data to NACC. Recruitment methods and sample characteristics varied across each ADC, representing a mixture of clinical- and community-based sampling.

### Baseline Versus 12-Month Diagnoses

For all analyses, participant clinical diagnostic groups were determined based on the 12-month follow-up examination to ensure that NP decline fell within the range of the appropriate diagnostic classifications (i.e., decline within normal range cognition, decline within no MCI range cognitive impairment, and decline within MCI range cognition).

### Regression-Based Norms for Neuropsychological Decline Using the Alzheimer’s Disease Neuroimaging Initiative Database

To avoid circularity in our investigation into the predictive utility of a neuropsychological marker for future dementia risk (i.e., NP decline), we first developed the NP decline marker using normative data from a reference sample in one dataset (the ADNI data) and then applied these norms to a separate test sample from another dataset (the NACC data). To avoid circularity and criterion contamination of clinical diagnosis by the neuropsychological markers themselves, all findings were also confirmed using progression from a CDR^®^ Dementia Staging Instrument score of 0 to a score of 0.5 or higher as the criterion measure, rather than clinical diagnosis.

The NP decline metric was operationalized by developing linear regression equations in a robustly normal reference sample from the ADNI database (n = 294). For this analysis, we used methods described in detail recently (Nation et al., 2019). Briefly, a robustly normal subset of cognitively normal older adults from the ADNI study was identified using criteria established by prior ADNI studies (Edmonds et al., 2015a): (1) participants were identified as cognitively normal on baseline ADNI assessment and (2) participants remained cognitively normal throughout the duration of their study participation.

Linear regression was used to model the relationship between baseline performance on a neuropsychological test and 12-month follow-up performance on the same test using longitudinal ADNI study data. Neuropsychological tests included Wechsler Memory Scale – Revised (WMS-R) Logical Memory Story A immediate (Logical Memory I) and delayed (Logical Memory II) free recall, Trails A and B, and Animals and Vegetables. Specific neuropsychological tests were chosen based on the overlap between ADNI neuropsychological tests (reference sample) and tests available in NACC (test sample), as well as the desire to evaluate a balance of 2 tests per domain across domains relevant to dementia risk, including memory, attention/executive function, and language (Bondi et al., 2008). Scores from Trails A and B exhibited significant skewness, which was corrected by log transformation. These scores were also inverted (i.e., multiplied by -1) such that higher scores indicate better performance, consistent with all other neuropsychological measures.

The result of linear regression analyses evaluating baseline test performance as a predictor of 12-month follow-up test performance produced linear regression equations that represent the relationship between baseline and 12-month test performance in a robustly normal sample (refer to Supplementary Table 1 for details regarding linear regression parameters in the robustly normal ADNI sample). These regression-based norms were developed for the purpose of calculating standardized scores for NP decline over 12 months relative to normative expectations (as in Nation et al., 2019). This study sought to apply these ADNI-derived regression-based norms to a test sample from the NACC database to determine whether the resulting NP decline metric may be of value in predicting future dementia among older adults who were cognitively normal or mildly impaired during their first 12 months of neuropsychological follow-up.

### Applying Regression-Based Norms From Alzheimer’s Disease Neuroimaging Initiative to the National Alzheimer’s Coordinating Center Database

In this study, the linear regression equations developed in the robustly normal sample from ADNI (refer to earlier) were used to quantify NP decline scores for all eligible participants in the NACC database with a baseline clinical consensus diagnosis of normal cognition, impaired without MCI or MCI. Below, Eq. 1 shows the template for the normative regression equations developed from raw scores in robustly normal participants in ADNI and used to calculate the predicted 12-month performance for each test for NACC participants (Eqs 2–7).

The NP decline metric was calculated as previously described using three steps (Nation et al., 2019), namely, (1) baseline NACC participant raw scores on neuropsychological testing (refer to earlier for battery) were entered into the linear regression equations (Eqs 2–7) developed using robustly normal participants from ADNI. Linear regression equations used baseline raw scores to calculate the predicted 12-month performance on each neuropsychological test based on normative expectations from the ADNI subsample. (2) For each participant, the predicted 12-month performance based on the regression-based norms from ADNI was then subtracted from the actual 12-month performance for each neuropsychological test, and the resulting discrepancy between the 12-month predicted performance and the actual performance was divided by the standard error of the estimate for each linear regression equation corresponding to each neuropsychological test (refer to Eq. 8 below). (3) The standardized scores were averaged across all 6 neuropsychological test scores to create the NP decline z-score.

As shown in Eq. 8, NP decline raw scores were standardized by dividing the standard error of the estimate (S_y.x_) drawn from each regression equation (Crawford and Howell, 1998; Crawford and Garthwaite, 2006): Eq. 2 S_y.x_ = 2.7730, Eq. 3 S_y.x_ = 3.1780, Eq. 4 S_y.x_ = 0.1009, Eq. 5 S_y.x_ = 0.1374, Eq. 6 S_y.x_ = 4.0650; and Eq. 7 S_y.x_ = 3.2700.


(1) P⁢r⁢e⁢d⁢i⁢c⁢t⁢e⁢d⁢s⁢c⁢o⁢r⁢e=i⁢n⁢t⁢e⁢r⁢c⁢e⁢p⁢t+(c⁢o⁢e⁢f⁢f⁢i⁢c⁢i⁢e⁢n⁢t×b⁢a⁢s⁢e⁢l⁢i⁢n⁢e⁢s⁢c⁢o⁢r⁢e)

(2) P⁢r⁢e⁢d⁢i⁢c⁢t⁢e⁢d⁢L⁢o⁢g⁢i⁢c⁢a⁢l⁢M⁢e⁢m⁢o⁢r⁢y⁢I=6.883+(0.595×b⁢a⁢s⁢e⁢l⁢i⁢n⁢e⁢L⁢o⁢g⁢i⁢c⁢a⁢l⁢M⁢e⁢m⁢o⁢r⁢y⁢I)

(3) P⁢r⁢e⁢d⁢i⁢c⁢t⁢e⁢d⁢L⁢o⁢g⁢i⁢c⁢a⁢l⁢M⁢e⁢m⁢o⁢r⁢y⁢I⁢I=4.810+(0.680×b⁢a⁢s⁢e⁢l⁢i⁢n⁢e⁢L⁢o⁢g⁢i⁢c⁢a⁢l⁢M⁢e⁢m⁢o⁢r⁢y⁢I⁢I)

(4) PredictedTrailsAlog=[0.589+(0.598×baselineTrailsAlog)]×-1

(5) PredictedTrailsBlog=[0.656+(0.643×baselineTrailsBlog)]×-1

(6) P⁢r⁢e⁢d⁢i⁢c⁢t⁢e⁢d⁢A⁢n⁢i⁢m⁢a⁢l⁢s=8.410+(0.623×b⁢a⁢s⁢e⁢l⁢i⁢n⁢e⁢A⁢n⁢i⁢m⁢a⁢l⁢s)

(7) P⁢r⁢e⁢d⁢i⁢c⁢t⁢e⁢d⁢V⁢e⁢g⁢e⁢t⁢a⁢b⁢l⁢e⁢s=4.464+(0.687×b⁢a⁢s⁢e⁢l⁢i⁢n⁢e⁢V⁢e⁢g⁢e⁢t⁢a⁢b⁢l⁢e⁢s)

(8) $$N\,P\,d\,e\,c\,l\,i\,n\,e\,s\,u\,b\,t\,e\,s\,t\,\text{z}=\frac{a\,c\,t\,u\,a\,l\,s\,c\,o\,r\,e-p\,r\,e\,d\,i\,c\,t\,e\,d\,s\,c\,o\,r\,e}{s\,t\,a\,n\,d\,a\,r\,d\,e\,r\,r\,o\,r\,o\,f\,t\,h\,e\,e\,s\,t\,i\,m\,a\,t\,e}$$

### Individual Test Scores Versus Overall Neuropsychological Decline Score

The examination of NP decline in individual test scores is beyond the scope of this study, which is focused instead on NP decline as a general cognitive decline factor assessed by multiple test scores. The use of single test scores to determine clinical status is also not advised, given the limited reliability of individual neuropsychological test scores for determining cognitive abnormality (Binder et al., 2009). Finally, our prior study developed an optimized cutoff value for NP decline based on the overall average NP decline across tests (Nation et al., 2019), providing an opportunity for cross-validation using the NACC data. For all these reasons, NP decline subtest z-scores were averaged to create a global NP decline score for all statistical analyses, as described earlier.

### Neuropsychological Decline Cutoff Values – Cross-Validation

The optimal cutoff values for NP decline in the ADNI study were previously determined by receiver operating characteristic (ROC) curve analysis. Results of the ROC curve analysis indicated an optimal NP decline z-score of -0.5808, corresponding approximately to the 28th percentile of the NP decline distribution (Nation et al., 2019). This z-score represents an optimal cutoff value for the NP decline metric in terms of predicting the development of dementia. It is a z-score of the distribution of NP decline, computed as predicted performance for normal aging subtracted from actual 12-month follow-up performance, and standardized by the standard error of the estimate. Cognitively normal older adults performing below this NP decline z-score at 12-month follow-up exhibited more rapid progression to dementia, relative to those above the cutoff value. This was regardless of demographic factors, biomarker status, or APOE4 carrier status (Nation et al., 2019). For cross-validation, this study used this same cutoff value derived from the ADNI study to determine dementia risk based on NP decline in the NACC sample.

### Statistical Analyses

All study variables were evaluated for departures from normality and potentially influential outliers. Trails A and B scores were log-transformed to improve normality for the purposes of linear regression models of NP decline (refer to Eqs 4, 5 above).

Participants were divided into groups based on the combination of their 12-month NACC clinical diagnostic status (cognitively normal, impaired without MCI, and MCI) and their final diagnostic status (no dementia vs. dementia). Participant groups were compared on their baseline demographic and clinical measures, including age, sex, and education using a 2 × 2 (diagnostic status × NP decline status) ANCOVA controlling for age, sex, and years of education, with *post-hoc* Bonferroni-corrected pairwise comparisons. Chi-squared analyses were used to compare the rate of future dementia by clinical diagnostic and NP decline status. Cox regression was used to evaluate the predictive value of NP decline in the overall sample and within each clinical diagnostic group, controlling for age, sex, and education.

## Results

Participant demographics and clinical data are presented in Table 1. Cognitively normal older adults with greater than expected 12-month NP decline (below-established cutoff value) were significantly more likely to develop dementia over all follow-up relative to those above the cutoff value χ^2^ (1, *N* = 4,692) = 55.02, *p* < 0.00001. Impaired without MCI participants with greater than expected 12-month NP decline (below-established cutoff value) were significantly more likely to ultimately develop dementia over all follow-up relative to those above the cutoff value χ^2^ (1, *N* = 470) = 4.78, *p* < 0.05. Similarly, MCI participants with greater than expected 12-month NP decline (below-established cutoff value) were significantly more likely to ultimately develop dementia over all follow-up relative to those above the cutoff value χ^2^ (1, *N* = 1,632) = 29.21, *p* < 0.00001.

**TABLE 1:** Participant demographics and clinical characteristics.

Demographics	Mean ± SD or n	Range or %	
Age (years)	74.01 ± 7.82	60-104	
Education (years)	15.52 ± 3.21	0-30	
Male to Female Ratio	2,618 to 4,176	38.5% male	
**NACC Diagnosis at 12-months**			
Normal Cognition	4,692	69.1%	
Impaired MCI−	470	6.9%	
Impaired MCI+	1,632	24.0%	
**Progression to dementia**			
Dementia at Follow up	764	11.2%	
Follow up (months)	58.97 ± 29.88	19-158	
**NACC diagnosis × NP decline**			**% Dementia conversion**
Normal/NP−	3,557	52.4%	4.2%
Normal/NP+	1,135	16.2%	10.0%
Impaired MCI−/NP−	308	4.5%	9.7%
Impaired MCI−/NP+	162	2.4%	16.7%
Impaired MCI+/NP−	738	10.9%	20.6%
Impaired MCI+/NP+	894	13.2%	32.6%

*MCI−, Mild Cognitive Impairment absent; MCI+, Mild Cognitive Impairment present; NP−, Neuropsychological Decline absent; NP+, Neuropsychological Decline present; SD, standard deviation; NACC, National Alzheimer’s Coordinating Center.*

**FIGURE 1:**
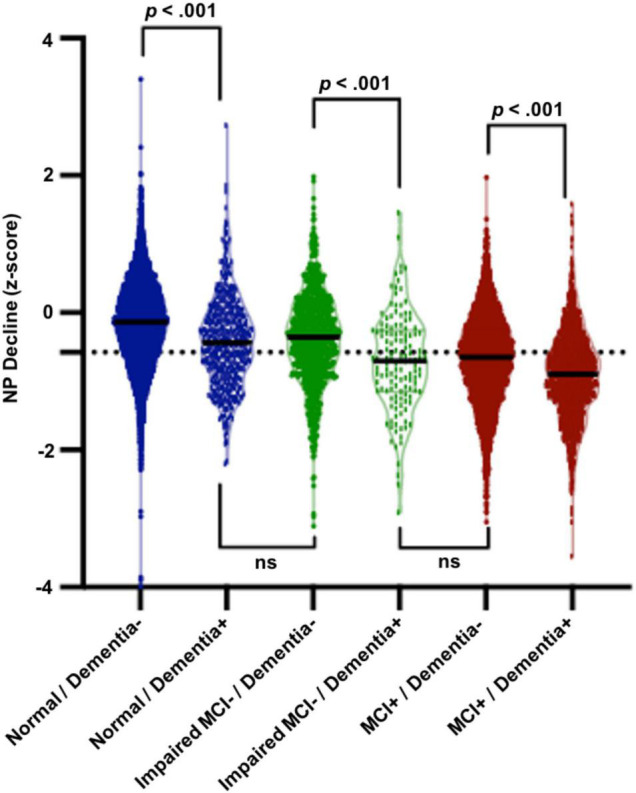
Results of 2 × 2 ANCOVA (baseline clinical diagnosis × dementia outcome). Cognitively normal older adults who ultimately developed dementia exhibited significantly worse NP decline (M = –0.46, SD = 0.57, range = 3.32) than those who did not develop dementia (M = –0.14, SD = 0.59, range = 3.98, *p* < 0.001) and did not significantly differ in NP decline from those who were impaired without MCI. Similarly, impaired without MCI participants who progressed to dementia displayed worse NP decline (M = –0.58, SD = 0.60, range = 2.55) than those who did not progress to dementia (M = –0.31, SD = 0.64, range = 2.50, *p* < 0.001) and did not significantly differ from those who were diagnosed with MCI. Finally, MCI participants who progressed to dementia exhibited significantly greater NP decline (M = –0.79, SD = 0.58, range = 2.59) than those who did not progress to dementia (M = –0.58, SD = 0.63, range = 2.35, *p* < 0.001).

Results of 2 × 2 ANCOVA (baseline clinical diagnosis × dementia outcome) with NP decline z-score as the dependent measure are presented in Figure 1. Cognitively normal older adults who ultimately developed dementia exhibited significantly worse NP decline than those who did not develop dementia (*p* < 0.001) and did not significantly differ in NP decline from those who were impaired without MCI. Similarly, impaired without MCI participants who progressed to dementia displayed worse NP decline than those who did not progress to dementia (*p* < 0.001) and did not significantly differ from those who were diagnosed with MCI. Finally, MCI participants who progressed to dementia exhibited significantly greater NP decline than those who did not progress to dementia (*p* < 0.001).

**FIGURE 2:**
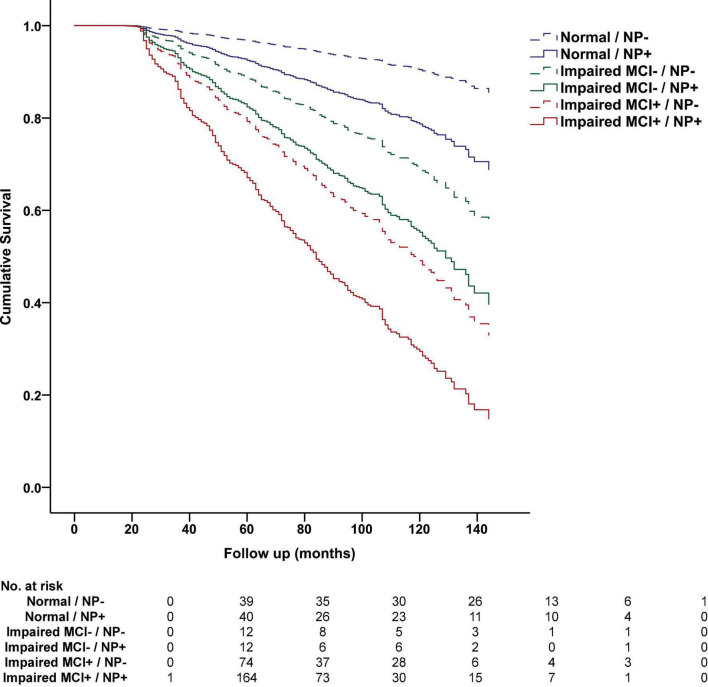
Progression to dementia stratified by cognitive status and NP decline status in the National Alzheimer’s Coordinating Center Database. Cumulative progression to dementia from Cox regression analysis is displayed and stratified by baseline NACC diagnosis, including Normal Cognition (Normal), Impaired without MCI (Impaired MCI−), Impaired with MCI (Impaired MCI+), and NP decline status at 12-month follow-up based on optimal cutoff values, including NP decline absent (NP−, above 28th percentile) and NP decline present (NP+, at or below 28th percentile). The table below displays the number of participants who progressed to dementia at each follow-up interval.

On longitudinal analysis, NP decline predicted future all-cause dementia in the total sample, after controlling for age, sex, and education, -2 log likelihood = 11,874.363, χ^2^ = 295.601.71, hazard ratio [HR] = 2.806, *p* < 0.001, and in the subset with normal cognition, -2 log likelihood = 3,776.938, χ^2^ = 40.842, HR = 2.006, *p* < 0.001, impaired without MCI diagnosis, -2 log likelihood = 574.928, χ^2^ = 14.891, HR = 2.465, *p* < 0.001, and impaired with MCI diagnosis, -2 log likelihood = 5,747.221, χ^2^ = 55.772, HR = 1.916, *p* < 0.001. Results of Cox regression analysis stratified by clinical diagnosis and NP decline status are presented in Figure 2.

## Discussion

Among older adults with a baseline diagnosis spanning the cognitively unimpaired-to-MCI spectrum, NP decline indicative of worse than expected 12-month follow-up performance was associated with an approximately 2-fold increase in risk for all-cause dementia at each follow-up, even after accounting for age, sex, and education. Thus, NP decline may represent a valuable adjunctive tool for risk stratification in both normal and mildly impaired older adults followed for at least 12 months. Frequently used diagnostic criteria for MCI and for cognitive decline in the context of Alzheimer’s disease rely heavily on subjective self-report and informant report to assess the presence of longitudinal decline (Jack et al., 2018), but subjective reports of cognitive change are influenced by psychiatric symptoms, personality traits, and other unrelated factors that may contribute to diagnostic error (Edmonds et al., 2014, Edmonds et al., 2018). The addition of an NP decline marker to the existing protocols could aid in the identification and recruitment of high-risk participants for clinical trials focusing on preclinical or MCI populations.

Many prospective studies of aging follow participants with annual or semi-annual neuropsychological exams, but these data are not always used to determine dementia risk. The NP decline approach presented earlier provides simple equations for standardizing the discrepancy between expected performance and actual performance at follow-up (Crawford and Garthwaite, 2006; Slick, 2006; Nation et al., 2019). The NP decline metric may be valuable in the context of these longitudinal aging studies since 12-month NP decline can be easily calculated to determine whether participants are showing worse than expected follow-up performance. Critically, participants showing NP decline beyond optimal cutoff values were at an increased risk for future dementia even if they were still performing within the normative range at 12-month follow-up. Clinicians often follow at-risk individuals on an annual or semi-annual basis, yielding serial neuropsychological data that can be easily evaluated using the provided equations and cutoff values for NP decline quantification.

Data from 12-month NP decline may help inform clinician judgments since decline beyond optimal cutoff values has now been linked to an approximately 2-fold increase in risk for dementia in two large longitudinal cohorts (Nation et al., 2019). Thus, there may be immediate value in terms of both research and clinical applications of the NP decline metric, allowing clinicians to gather further prognostic information beyond that obtained by the diagnosis of normal cognition or MCI. It is also important to note that even short-term practice effects (e.g., exams separated by 1 week) have also shown to be indicative of later cognitive decline (e.g., Duff et al., 2011). Practice effects across 1 week are related to diagnosis (Duff et al., 2008), prognosis (Duff et al., 2007, 2011), and treatment response (Duff et al., 2010), showing how the examination of these is another critical future direction of this work.

The potential application of NP decline analysis goes beyond any specific dementia etiology, but it should also be noted that recent research recommendations for the diagnosis of Alzheimer’s disease have emphasized the evaluation of serial cognitive test data to determine early or subtle cognitive decline (Jack et al., 2018). Although prior study has focused primarily on single exam methods for identifying older adults with subtle cognitive decline (Donohue et al., 2014; Edmonds et al., 2015b; Toledo et al., 2015), serial exams may be required in order to detect the earliest cognitive changes represented by a decline within normal range performance. The method employed in this study allows for quantification and standardization of longitudinal decline within normal range performance, which may better detect subtle cognitive changes related to an incipient neuropathological process. Numerous studies have emphasized the role of biomarkers in the stratification of dementia risk in cognitively unimpaired older adults (Jack et al., 2018), but other studies have shown that many older adults with biomarker abnormalities will never develop dementia (Ritchie et al., 2017). Combining sensitive preclinical neuropsychological instruments with preclinical biomarkers may aid in prognostic evaluation and treatment decision-making beyond information obtained through biomarker analysis alone (Nation et al., 2019).

Strengths of this study include the longitudinal analysis and large sample size. Limitations include the variable clinical follow-up and heterogeneity of NACC sampling methods that includes a mixture of studies from numerous sites with both clinical- and community-based studies. Furthermore, the NACC database has limited ethnic diversity, with NACC participants being largely Caucasian. However, of note, the NACC database does enroll participants with diverse medical history, including dementia of various etiologies, and this heterogeneity of NACC data benefits the generalizability of the study findings, particularly since the results coincided well with the recently published data from the more curated ADNI study sample (Nation et al., 2019). The use of neuropsychological test data to predict future dementia risk has also been criticized for circularity. Although neuropsychological test data can often be used to aid in the diagnosis of dementia in conjunction with other data, including measures of functional decline, informant reports, behavioral observations, and clinician judgments, this study evaluated the predictive value of neuropsychological markers in older adults with normal to mildly impaired cognitive function. Thus, neuropsychological markers may be useful prognostic instruments capable of stratifying future dementia risk even in patients with normative cognition, or only mild cognitive changes, with no functional decline or very minimal functional change. In this context, neuropsychological markers are not diagnostic of dementia, but rather they are prognostic indicators that may be of value in the detection of an incipient decline in neurocognitive function, potentially presaging the future development of major cognitive and functional impairments that characterize dementia. The use of cognitive data to predict dementia risk based on MCI diagnosis is a well-established practice (Petersen, 2011) that is no more circular than the use of neuropsychological markers to predict future dementia from an even earlier stage, as in this study. Just as MCI is a risk factor for dementia, NP decline is a risk factor for dementia. These risk factors are not circular. One of the most valuable aspects of NP decline is that it may be used in conjunction with MCI diagnosis, or even in cognitively unimpaired older adults, further stratifying and refining dementia risk assessment.

Additional research and development of methods for longitudinal analysis of serial neuropsychological exam data will improve our ability to determine patient cognitive trajectories, which will have major implications for neuropsychological research, clinical trials, and clinical practice in a variety of patient populations.

## Data Availability Statement

The original contributions presented in this study are included in the article/Supplementary Material, further inquiries can be directed to the corresponding author.

## Ethics Statement

Ethical review and approval was not required for the study on human participants in accordance with the local legislation and institutional requirements. Written informed consent for participation was not required for this study in accordance with the national legislation and the institutional requirements.

## Author Contributions

DN drafted the manuscript and acquired financial support for this manuscript. JH and DN developed and designed the study, conducted analyses and interpretation of the data, revised the manuscript, and approved the submitted version.

## Conflict of Interest

The authors declare that the research was conducted in the absence of any commercial or financial relationships that could be construed as a potential conflict of interest.

## Publisher’s Note

All claims expressed in this article are solely those of the authors and do not necessarily represent those of their affiliated organizations, or those of the publisher, the editors and the reviewers. Any product that may be evaluated in this article, or claim that may be made by its manufacturer, is not guaranteed or endorsed by the publisher.
