# Supplementary material for: Neuropsychological Decline Stratifies Dementia Risk in Cognitively Unimpaired and Impaired Older Adults
Source: Front Aging Neurosci. 2022 Jul 18;14:838459. doi: 10.3389/fnagi.2022.838459 (PMC9339652; doi:10.3389/fnagi.2022.838459)
Supplement: Supplementary file 1 [file Table_1.docx]

Supplementary Material

**Supplementary Table 1.** In the robustly normal sample from the Alzheimer’s Disease Neuroimaging Initiative (ADNI), we used the linear regression model (Eq. 1) assuming that baseline performance (*X*) can predict future performance (*Y*) using the intercept (*a*) and slope (*b*) of a robust normal sample with a random, normally distributed error term (*ε*). Linear regression models from the robust normal sample yielded equations predicting 12-month performance from baseline performance (Eq. 2-7).

| Eq. 1 | $Y=a+bX+\varepsilon$ |
| --- | --- |
| Eq. 2 | Predicted AVLT Trials 1-5 total = 13.214 + (0.720 × Baseline AVLT Trials 1-5 total) |
| Eq. 3 | Predicted AVLT Trials 6-7 average = 3.270 + (0.645 × Baseline AVLT Trials 6-7 average) |
| Eq. 4 | Predicted Trails A (log) = 0.589 + (0.598 × Baseline Trails A (log)) × -1 |
| Eq. 5 | Predicted Trails B (log) = 0.656 + (0.643 × Baseline Trails B (log)) × -1 |
| Eq. 6 | Predicted Animal Fluency = 8.410 + (0.623 × Baseline Animal Fluency) |
| Eq. 7 | Predicted Vegetable Fluency = 4.464 + (0.687 × Baseline Vegetable Fluency) |
